# Promising efficacy of immune checkpoint inhibitor plus chemotherapy for thoracic SMARCA4-deficient undifferentiated tumor

**DOI:** 10.1007/s00432-023-04806-y

**Published:** 2023-04-28

**Authors:** Ying Lin, Bo Yu, Haifeng Sun, Hongyu Zhang, Zhihuang Hu, Yao Zhang, Zhenhua Wu, Si Sun, Xinmin Zhao, Hui Yu, Xianghua Wu, Yuan Li, Jialei Wang, Huijie Wang

**Affiliations:** 1 Department of Thoracic Medical Oncology, Fudan University Shanghai Cancer Center, Shanghai, 200032 China; 2 Department of Oncology, Shanghai Medical College, Fudan University, No. 270, Dong’an Road, Shanghai, 20032 China; 3 Third Department of Medical Oncology, Shaanxi Provincial Cancer Hospital Affiliated to Medical College of Xi’an Jiaotong University, Xi’an, 710065 Shaanxi China; 4 Department of Thoracic Surgery, Zhongshan Hospital, Fudan University, Shanghai, 200032 China; 5 Department of Pathology, Fudan University Shanghai Cancer Center, Shanghai, 200032 China

**Keywords:** Thoracic SMARCA4-deficient undifferentiated tumor, SMARCA4-deficient thoracic sarcomatoid tumor, SMARCA4-deficient thoracic sarcoma, Immune checkpoint inhibitor, LRP1B

## Abstract

**Purpose:**

Thoracic SMARCA4-deficient undifferentiated tumor (SD-UT) is a highly aggressive disease that is nosologically related to but distinct from SMARCA4-deficient non-small cell lung cancer (SD-NSCLC). No standard treatment guidelines were established for SD-UT. This research explored the efficacy of different treatments in SD-UT, and the prognostic, clinicopathologic and genomic difference between SD-UT and SD-NSCLC.

**Materials and methods:**

Information of 25 SD-UT and 22 SD-NSCLC patients diagnosed and treated in Fudan University Shanghai Cancer Center from January, 2017 to September, 2022 was analyzed.

**Results:**

SD-UT was similar to SD-NSCLC in characteristics of onset age, male prevalence, heavy smoking history and metastatic pattern. SD-UT showed a rapid relapse pattern after radical therapy. For Stage IV SD-UT patients, immune checkpoint inhibitor (ICI) plus chemotherapy significantly improved median progression-free survival (PFS) compared to traditional chemotherapy as first-line treatment (26.8 *vs.* 2.73 months, *p* = 0.0437), while objective response rates of two arms were comparable (71.4% *vs.* 66.7%). No significant survival differences were observed between SD-UT and SD-NSCLC under similar treatment settings. SD-UT or SD-NSCLC patients receiving ICI in the first line had significantly prolonged OS than those with ICI in the latter lines or without ICI treatment throughout clinical courses. Genetic study found frequent SMARCA4, TP53 and LRP1B mutations in SD-UT.

**Conclusion:**

To the best of our knowledge, this is the largest series to date to compare the efficacy of ICI-based treatment to chemotherapy and document frequent mutations of LRP1B in SD-UT. ICI plus chemotherapy is an effective strategy for Stage IV SD-UT.

**Supplementary Information:**

The online version contains supplementary material available at 10.1007/s00432-023-04806-y.

## Introduction

SMARCA4 (BRG1) is one of the key components of the evolutionarily conserved switch/sucrose-nonfermentable (SWI/SNF) chromatin remodeling complex which plays important roles in transcription, differentiation and DNA repair (Mardinian et al. 2021). Recently, genetic alterations and protein aberrant expression of subunits of SWI/SNF complex including SMARCA4, SMARCA2 and SMARCB1 have been reported in several cancers, and been found to be associated with responses to immune checkpoint inhibitor (ICI) (Tian et al. 2023; Wanior et al. 2021). In thoracic tumors, loss of SMARCA4 expression occurs in approximately 5% of non-small cell lung cancer (NSCLC) and was associated with more aggressive clinical behavior (Herpel et al. 2017; Velut et al. 2022).

Another thoracic tumor harboring genetic alterations and protein aberrant expression of SMARCA4 is SMARCA4-deficient undifferentiated tumor (SD-UT), a recently recognized entity. SD-UT is characterized by undifferentiated rhabdoid morphology and loss of expression of SMARCA4 (Loarer et al. 2015; Perret et al. 2019; Sauter et al. 2017; Yoshida et al. 2017). It was found to be transcriptionally distinct from conventional NSCLC (Le Loarer et al. 2015; Perret et al. 2019; Sauter et al. 2017; Yoshida et al. 2017). Therefore, it was previously proposed as a type of thoracic sarcoma and named SMARCA4-deficient thoracic sarcoma (SD-TS) or SMARCA4-deficient thoracic sarcomatoid tumor (SD-TST) (Le Loarer et al. 2015). However, recent study found that SD-UT has a closer kinship with SD-NSCLC than with sarcomas, considering partial presence of NSCLC components in SD-UT, focal expression of NSCLC lineage markers on immunohistochemistry (IHC), common heavy smoking history, and mutation characteristics more similar to NSCLC than to sarcomas (Rekhtman et al. 2020). However, SD-UT is distinct from SD-NSCLC and not otherwise specified NSCLC with clinical presentations of larger and compressive primary tumor size, higher incidence at younger patients (30–50 years old), and significantly worse prognosis with median overall survival of about 6 months (Le Loarer et al. 2015; Luo et al. 2022; Perret et al. 2019; Rekhtman et al. 2020; Sauter et al. 2017; Yoshida et al. 2017). Therefore, these two types of thoracic tumors harboring loss of SMARCA4 should be viewed as two related but different entity. In the recent fifth edition of World Health Organization classification of Thoracic Tumors, the entity was recognized distinctively and renamed SD-UT (Nicholson et al. 2022).

As for treatment guidelines, SD-NSCLC is treated in accordance with guidelines of NSCLC, but several studies indicated detrimental effect of SMARCA4 alteration in prognosis (Alessi et al. 2021; Herpel et al. 2017; Velut et al. 2022). However, no clear guidelines have been established for the treatment of SD-UT. Previous treatment of traditional cytotoxic agents, including regimens for sarcomas showed very limited responses (Perret et al. 2019; Sauter et al. 2017; Yoshida et al. 2017). Results regarding efficacy of immunotherapy in SD-UT were controversial (Gantzer et al. 2022; Henon et al. 2019; Iijima et al. 2020; Kawachi et al. 2021; Shinno et al. 2022; Takada et al. 2019; Utsumi et al. 2022). On one hand, SD-UT was found to carry high tumor mutation burden, and benefited from either ICI monotherapy or combination therapy (Henon et al. 2019; Iijima et al. 2020; Kawachi et al. 2021; Rekhtman et al. 2020; Shinno et al. 2022; Takada et al. 2019; Utsumi et al. 2022). One the other hand, SD-UT was found to have low immune cell infiltrates and showed limited response to ICI (Gantzer et al. 2022). Due to the rarity of SD-UT, above studies had limited sample sizes. Therefore, no conclusions could be drawn. In addition, the prognostic difference between metastatic SD-NSCLC and SD-UT under different treatment settings remains unclear.

To further investigate efficacy of different treatments in SD-UT, explore the prognostic difference, clinicopathologic and genomic features of SD-NSCLC and SD-UT, we conducted a comprehensive retrospective study.

## Materials and methods

### Patient population and data collection

Medical records from January, 2017 to September, 2022 of Fudan University Shanghai Cancer Center (FUSCC) was retrospectively searched. Patients diagnosed in FUSCC with SD-UT or SD-TST, or SD-TS, or SD-NSCLC were identified. Patients were staged according to the American Joint Committee on Cancer Staging Manual (8th Edition) of NSCLC. Last follow-up time was March 10th 2023. Results of molecular testing performed in our center or in other institutes were collected.

### Efficacy and safety assessments

Disease-free survival (DFS) was defined as the time from primary radical treatment to the date of relapse. Progression-free survival (PFS) was defined as the time from treatment initiation to date of disease progression or death of any cause, regardless of whichever occur first. Overall survival (OS) was defined as the time from treatment initiation to the date of death of any cause. The objective response rate (ORR) was defined as the proportion of patients with complete response (CR) or partial response (PR) based on Response Evaluation Criteria in Solid Tumor criteria (ver. 1.1).

### Statistical analysis

Survival was calculated by the Kaplan–Meier method and the subgroup comparisons were evaluated using the log-rank test. All statistical analyses were performed using SPSS ver. 19 (SPSS Inc., Chicago, IL). All statistical tests were two tailed and *p* < 0.05 was considered as statistically significant.

## Results

### Clinicopathologic characteristics of SD-UT and SD-NSCLC.

A total of 47 patients were diagnosed with SD-UT or SD-NSCLC in FUSCC from January, 2017 to September, 2022, including 25 SD-UT patients and 22 SD-NSCLC patients. Characteristics were summarized in Table 1. Different from previous reports (Perret et al. 2019; Rekhtman et al. 2020; Sauter et al. 2017), the mean age of onset was similar between SD-UT and SD-NSCLC in our cohort. Patients were exclusively male. Mean packyear smoking history was 38 and 40, respectively, in SD-UT and SD-NSCLC. More patients were diagnosed with stage IV disease in SD-UT group than SD-NSCLC group, although the difference was not statistically significant. Metastatic pattern of SD-UT was similar to SD-NSCLC, with distant lymph nodes, adrenal glands, parietal pleura and liver the most common metastatic sites in SD-UT. Mean tumor mutational burden (TMB) in SD-UT was numerically but not significantly higher than that in SD-NSCLC (18.6 *vs.* 8.7/Mb, *p* = 0.087).

**Table 1:** Characteristics of thoracic SMARCA4-deficient undifferentiated tumor (SD-UT) and SMARCA4-deficient non-small cell lung cancer (SD-NSCLC)

Characteristic	SD-UT (*n* = 25)	SD-NSCLC (*n* = 22)	*P* value
Age, mean (range)	62 (38–80)	58 (37–77)	0.119
< 50	3 (12%)	4 (18.2%)	
50–60	5 (20%)	8 (36.4%)	
60–70	14 (56%)	8 (36.4%)	
≥ 70	3 (12%)	2 (9.1%)	
Gender			
Male	25 (100%)	22 (100%)	> 0.999
Female	0 (0%)	0 (0%)	
Smoking status, mean (pack-years)	38	40	0.865
Never smokers	0 (0%)	1 (4.5%)	
≥ 20	11 (44%)	11 (50%)	
< 20	2 (8%)	4 (18.2%)	
Unknown	12 (48%)	6 (27.3%)	
Stage at diagnosis			
I–III	9 (36%)	13 (59.1%)	0.181
IV	14 (56%)	9 (40.9%)	
Unknown	2 (8%)	0 (0%)	
Common metastatic sites throughout patients’ clinical course			/
Distant lymph nodes	7 (28%)	9 (40.9%)	
Adrenal gland	7 (28%)	7 (31.8%)	
Parietal pleura	5 (20%)	6 (27.3%)	
Liver	4 (16%)	3 (13.6%)	
Bone	3 (12%)	6 (27.3%)	
Lung	3 (12%)	4 (18.2%)	
Chest wall	3 (12%)	2 (9.1%)	
Soft tissue	1 (4%)	4 (18.2%)	
Brain	2 (8%)	2 (9.1%)	
Tumor mutational burden (mean/Mb)	18.6 (*n* = 5)	8.7 (*n* = 4)	0.087
PD-L1			/
Negative	1 (4%)	5 (22.7%)	
1 ~ 49%	2 (8%)	5 (22.7%)	
≥ 50%	1 (4%)	2 (9.1%)	
Untest	21 (84%)	10 (45.5%)	

### Rapid relapse pattern and potential efficacy of ICI as neoadjuvant therapy in SD-UT with radical treatment

**Fig. 1:**
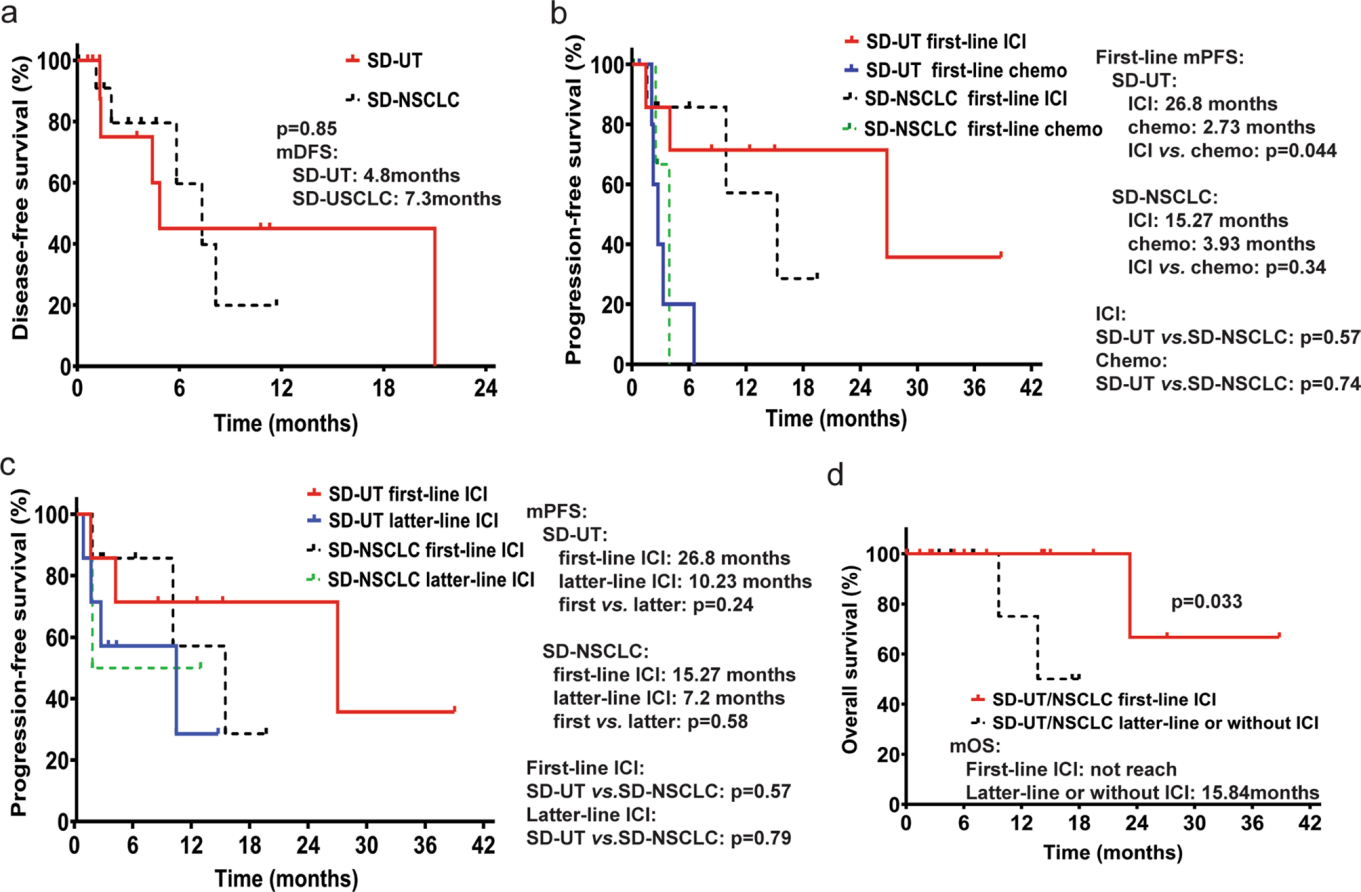
Kaplan–Meier plots of survival in patients with thoracic SMARCA4-deficient undifferentiated tumor (SD-UT) and SMARCA4-deficient non-small cell lung cancer (SD-NSCLC). **a** Kaplan–Meier plot of disease-free survival in SD-UT and SD-NSCLC patients. **b** Kaplan–Meier plots of progression-free survival in Stage IV SD-UT and SD-NSCLC patients receiving immune checkpoint inhibitor (ICI) or chemotherapy as first-line treatment. **c** Kaplan–Meier plots of progression-free survival in Stage IV SD-UT and SD-NSCLC patients receiving ICI in first-line treatment or in latter-line treatment. **d** Kaplan–Meier plots of overall survival in Stage IV SD-UT/NSCLC patients receiving ICI as first-line treatment, and patients receiving ICI in latter-line treatment or without ICI treatment. *mDFS* median disease-free survival; *mPFS* median progression-free survival; *mOS* median overall survival

Clinical summary of patients who underwent radical treatments is presented in Table 2. A total of 11 SD-UT and 12 SD-NSCLC patients underwent radical treatments. Median DFS was slightly but not statistically significantly shorter in SD-UT than SD-NSCLC (4.8 vs. 7.3 months), indicating a rapid relapse pattern, and ineffectiveness of adjuvant therapy (Fig. 1a). Notably, one patient was diagnosed with SD-NSCLC of left lung and SD-UT in left adrenal gland oligometastatic site, indicating close kinship between two diseases. One patient of SD-UT received neoadjuvant therapy of pembrolizumab plus nab-paclitaxel and carboplatin, followed by radical surgery. Pathology exam showed pathologic complete response in primary tumor region and lymph nodes.

**Table 2:** Clinical summary and recurrence pattern of SMARCA4-deficient undifferentiated tumor (SD-UT) and SMARCA4-deficient non-small cell lung cancer (SD-NSCLC) after radical treatments

Characteristic	SD-UT (*n* = 11)	SD-NSCLC (*n* = 12)
pTNM		
I	3 (27.3%)	5 (41.7%)
II	2 (18.2%)	1 (8.3%)
III	4 (36.4%)	6 (50%)
IV	2 (18.2%, oligometastasis)	0 (0%)
Treatment		
Surgery	10 (90.9%)	10 (83.3%)
Concurrent chemoradiation	1 (9.1%)	2 (16.7%)
Perioperative treatment		
Neoadjuvant therapy	1 (9.1%)	0 (0%)
Adjuvant chemotherapy	4 (36.4%)	6 (50%)
Adjuvant radiotherapy	1 (9.1%)	0 (0%)
Recurrent and metastatic sites after radical treatments	*n* = 5	*n* = 5
Regional lymph nodes	2 (40%)	0 (0%)
Distant lymph nodes	2 (40%)	1 (20%)
Parietal pleura	2 (40%)	2 (40%)
Chest wall	1 (20%)	1 (20%)
Liver	1 (20%)	1 (20%)
Adrenal gland	1 (20%)	4 (80%)
Skin and soft tissue	1 (20%)	2 (40%)
Brain	1 (20%)	0 (0%)
Pancreas	1 (20%)	0 (0%)
Bone	0 (0%)	2 (40%)
Lung	0 (0%)	1 (20%)
Kidney	0 (0%)	1 (20%)
Peritoneum	0 (0%)	1 (20%)

### ICI-based therapy was significantly more effective than traditional chemotherapy in Stage IV SD-UT, and no prognostic disparity was observed between SD-UT and SD-NSCLC

**Fig. 2:**
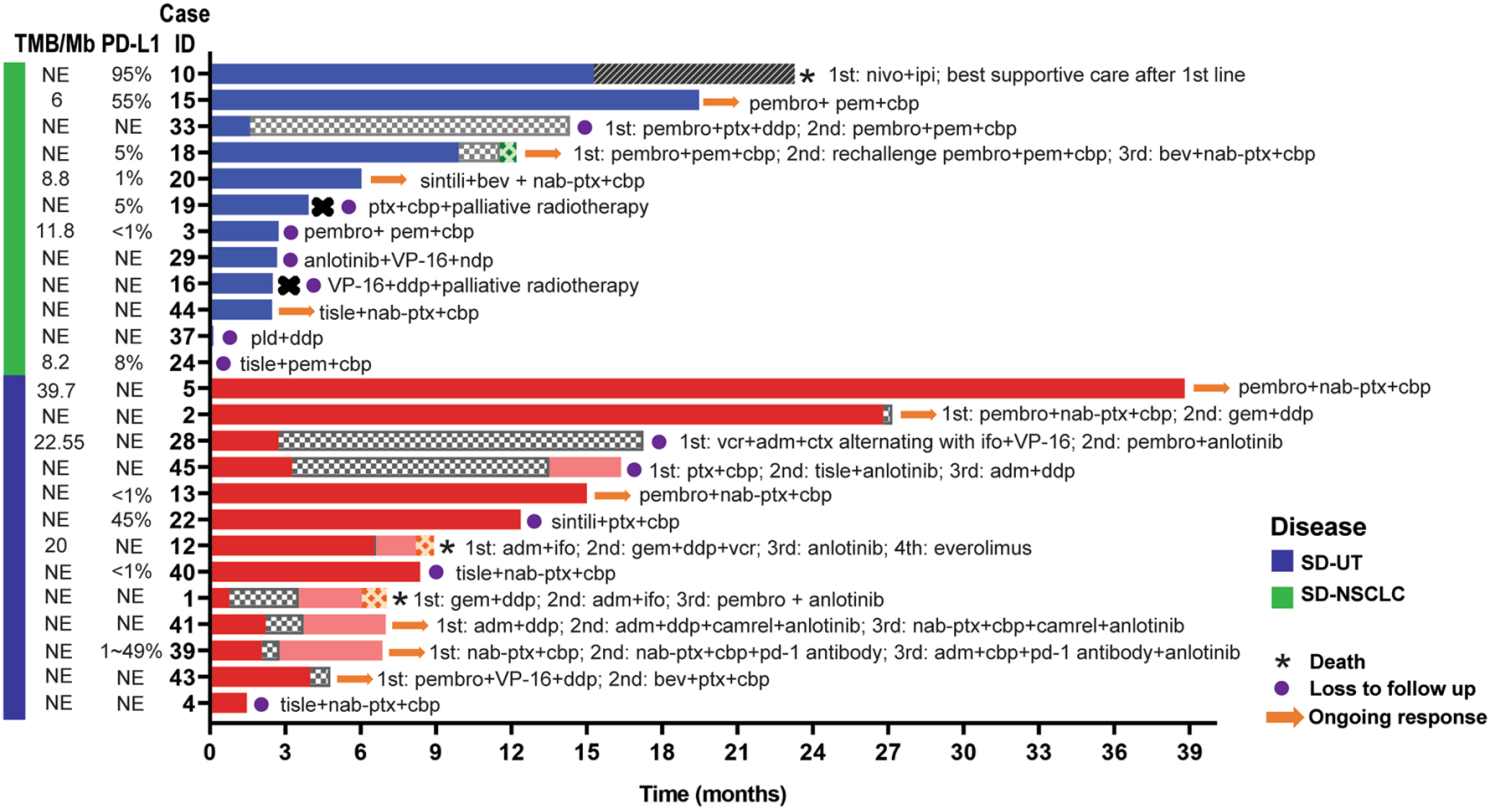
Swimming plot of treatments and clinical information for patients with Stage IV thoracic SMARCA4-deficient undifferentiated tumor (SD-UT) and SMARCA4-deficient non-small cell lung cancer (SD-NSCLC). The symbols on each bar indicated the treatment and living status. *1st* first line; *2nd* second line; *3rd* third line; *4th* fourth line; *nivo* nivolumab; *ipi* ipilimumab; *pembro* pembrolizumab; *pem* pemetrexed; *cbp* carboplatin; *ptx* paclitaxel; *ddp* cisplatin; *bev* bevacizumab; *nab-ptx* nab-paclitaxel; *sintili* sintilimab; *VP-16* etoposide; *ndp* nedaplatin; *tisle* tislelizumab; *pld* pegylated liposomal doxorubicin; *gem*: gemcitabine; *ctx* cyclophosphamide; *adm* doxorubicin; *ifo* ifosfamide; *vcr* vincristine; *camrel* camrelizumab

A total of 13 Stage IV SD-UT and 12 Stage IV SD-NSCLC were treated in our center. As for first-line therapy, ORRs of ICI-based therapy and chemotherapy arms were similar (71.4% *vs.* 66.7%, all PR) in SD-UT. A significant improvement in median PFS (mPFS) was observed in ICI-based therapy arm than chemotherapy arm as first-line therapy in SD-UT subgroup (*p* = 0.0437, Fig. 1b). In SD-NSCLC subgroup, mPFS was obviously improved in ICI-based arm than chemotherapy arm, although the difference is not statistically significant, probably due to small sample size (*p* = 0.148, Fig. 1b). Swimming plot and clinical information were shown in Fig. 2. ICI plus chemotherapy was the most common combination in our cohort, with the most common cytotoxic drugs being paclitaxel/nab-paclitaxel plus platinum in SD-UT subgroup, and paclitaxel/nab-paclitaxel or pemetrexed plus platinum in SD-NSCLC subgroup.

A total of 24 patients received at least one line of ICI-based treatment throughout their clinical courses. Although not statistically significant, the mPFS in patients treated with ICI in the first line was numerically longer than that in patients treated with ICI in second or latter line, both in SD-UT and SD-NSCLC subgroups (Fig. 1c). 2 patients with SD-UT who did not have ICI in first-line treatment received PD-1 antibody plus anlotinib, a multitarget tyrosine kinase inhibitor targeting tumor angiogenesis, as second-line therapy, and achieved long-time disease control of over 6 months (Fig. 2).

Regarding the prognostic disparity, no significant survival differences were observed in PFS between SD-UT and SD-NSCLC of under same treatment settings (Fig. 1b, c).

As for benefits in OS, SD-UT or SD-NSCLC patients receiving ICI-based therapy in the first line had significantly prolonged OS than those with ICI-based therapy in the latter lines or without ICI treatment throughout clinical courses (Fig. 1d). Kaplan–Meier curves of OS in all patients were showed in Supplementary Fig. 1.

Our results showed that ICI combination therapy was more effective than traditional chemotherapy in the first-line treatment of Stage IV SD-UT and SD-NSCLC, and it may achieve optimal efficacy used in front line than latter lines. No prognostic disparity was observed between SD-UT and SD-NSCLC.

### Genomic alterations in SD-UT and SD-NSCLC

**Fig. 3:**
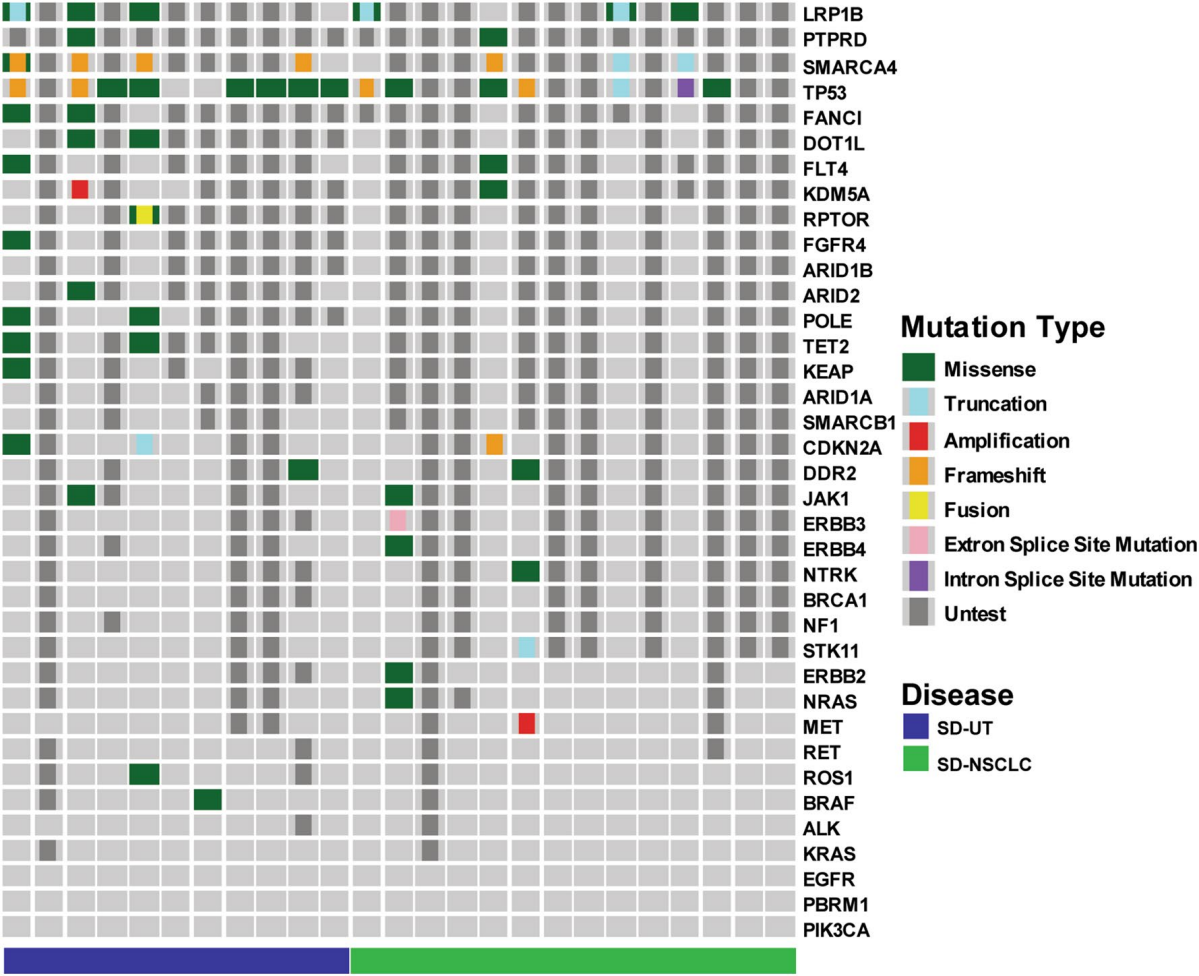
Genetic alterations of thoracic SMARCA4-deficient undifferentiated tumor (SD-UT) and SMARCA4-deficient non-small cell lung cancer (SD-NSCLC). A column represents a case and each row represents a gene

Results of molecular testing were available in 25 patients, including 11 SD-UT and 14 SD-NSCLC (Fig. 3). Most common mutated genes were SMARCA4, TP53 and LRP1B. SMARCA4 was sequenced in 9 patients, and 7 of them carried frameshift or truncated mutations. Common LRP1B mutations were reported for the first time in our knowledge in SD-UT. Of the 6 patients with LRP1B mutations, 3 carried truncated mutation, indicating functional damage of LRP1B. Genetic alterations in key NSCLC driver genes were not found. However, genes reported to be frequently mutated in SD-UT and SD-NSCLC, including KRAS, KEAP1, STK11 and NF1, were less frequently mutated in our cohort.

## Discussion

To our knowledge, this is the largest series to date to explore the survival benefit of ICI comparing to traditional chemotherapy in SD-UT, and the prognostic disparity between SD-NSCLC and SD-UT under different treatment settings.

Inactivating mutations and loss of expression of SMARCA4 have been implicated in thoracic tumors. Molecular analysis revealed SD-UT as a designated entity with highly aggressive behavior, extremely poor prognosis, and close relationship with SD-NSCLC (Le Loarer et al. 2015; Nicholson et al. 2022; Perret et al. 2019; Rekhtman et al. 2020; Sauter et al. 2017; Yoshida et al. 2017). In our study, we found one patient with SD-NSCLC in primary site and SD-UT in metastatic site, also indicating a link between SD-UT and NSCLC. In our cohort, patients were exclusively male in both SD-NSCLC and SD-UT subgroup, while previous reports contained a minority of female patients (Perret et al. 2019; Rekhtman et al. 2020; Sauter et al. 2017; Yoshida et al. 2017).

For limited-stage patients undergoing radical treatments, rapid relapse pattern was observed in SD-UT, suggesting ineffectiveness of perioperative treatments. Surprisingly, 1 patient in our cohort underwent neoadjuvant therapy of pembrolizumab plus nab-paclitaxel and carboplatin for 2 cycles and achieved pathologic complete response after radical surgery and stay tumor-free after 10-month follow-up. Consistently, there is a recent report about a patient diagnosed of SD-UT with vertebral and chest wall invasion undergoing surgery after 6 cycles of atezolizumab, bevacizumab, paclitaxel and carboplatin treatment and staying recurrence-free after 9-month follow-up (Kunimasa et al. 2021). These results indicated the potential efficacy of ICI combination therapy as perioperative treatment for SD-UT, but survival benefit needs further investigation.

For metastatic patients, different treatments were administrated by physicians’ choice. ICI-based treatment significantly improved PFS than chemotherapy in the first-line treatment of SD-UT, although ORR were similar between ICI and chemotherapy arm.

The median PFS was longer in patients receiving ICI as first-line therapy than as latter-line therapy in both SD-UT and SD-NSCLC. Patients of SD-UT and SD-NSCLC receiving ICI-based regimen as first-line treatment had significantly longer median OS than those having ICI-based regimen in latter line or no ICI treatment throughout clinical courses. These results indicated the promising efficacy of ICI in metastatic SD-UT, and its optimal effect may be achieved when used early in clinical courses.

However, some studies showed limited efficacy of ICI in SD-UT. Gantzer et al. reported that SD-UT mostly had an immune-desert tumor microenvironment (TME) (Gantzer et al. 2022). Only 1 out of 4 patients in Gantzer’s cohort who turned out to have immune-rich TME responded to ICI (Gantzer et al. 2022). In Gantzer’s cohort, patients received ICI treatment without chemotherapy (Gantzer et al. 2022). In our cohort, ICI was combined with chemotherapy when used in SD-UT patients. Combination of ICI and chemotherapy is an important strategy to improve efficacy (Heinhuis et al. 2019). Chemotherapy may improve the immunological environment and therefore enhance the antitumor response of ICI, even in immune-low profile tumor (Heinhuis et al. 2019). ICI combined with platinum-based chemotherapy has been proved to be a successful regimen in NSCLC (Gandhi et al. 2018; Socinski et al. 2018). As seen in our cohort and several previous reports, the strategy of ICI plus platinum-based chemotherapy showed promising efficacy in SD-UT, even in PD-L1-negative patients (Henon et al. 2019; Kawachi et al. 2021; Shinno et al. 2022). Another potential benefit of combing chemotherapy with ICI is rapid disease control during the first few weeks, which is also an important strategy in NSCLC (Paz-Ares et al. 2021). This is even more important for SD-UT because they usually present with large and compressive mass, and are in urgent need of symptom relief (Perret et al. 2019; Rekhtman et al. 2020; Sauter et al. 2017; Yoshida et al. 2017). Therefore, theoretically speaking, the combination of ICI and chemotherapy followed by ICI maintenance would provide the benefit of rapid symptom palliation and long-term survival. In contrary, ICI alone had limited efficacy in immune-low profile tumor. The difference in treatment strategy may partially explain the unsatisfying results in Gantzer’s study. However, no direct comparison of ICI monotherapy and ICI plus chemotherapy has been done in SD-UT, which nominates further studies. As for prognostic discrepancy, similar prognosis was observed between metastatic SD-UT and SD-NSCLC when they were treated in similar treatment settings.

In our study, we observed that ICI plus anlotinib, a multitarget tyrosine kinase inhibitor targeting tumor angiogenesis, was beneficial for some patients as second or latter line therapy. ICI combined with anti-angiogenesis has potential synergistic effects in NSCLC and may serve as a chemo-free strategy (Manegold et al. 2017). Further studies are needed to investigate the efficacy of ICI plus anti-angiogenesis in SD-UT.

As for genetic alteration characteristics, no alterations in established or putative NSCLC driver genes were found in SD-UT, which is consistent with previous reports (Kawachi et al. 2021; Rekhtman et al. 2020). No driver gene alterations were found in SD-NSCLC, either. 7 of 9 patients with SMARCA4 detected carried frameshift or truncated mutations, which indicates a relationship between genetic alteration and protein loss of SMARCA4. In addition, lack of SMARCA4 mutations in the other 2 patients suggests other mechanisms underlying protein loss of SMARCA4. TP53 and LRP1B mutations were also frequently mutated. TP53 is a well-known tumor suppressor and its mutations were frequently found in SD-UT in previous reports (Kawachi et al. 2021; Rekhtman et al. 2020). Frequent LRP1B mutations in SD-UT are reported for first time to our knowledge. LRP1B encodes low-density lipoprotein receptor-related protein 1b, and its mutations were recently found to be associated with improved efficacy of ICI in NSCLC (He et al. 2023; Yu et al. 2022). Therefore, the role of LRP1B and the impact of its mutations on SD-UT need further study. Contrary to previous reports (Kawachi et al. 2021; Rekhtman et al. 2020), we observed low frequency of STK11, KEAP1, and KRAS mutations in SD-UT and SD-NSCLC. Whether this difference was due to small sample size or race difference needs further study.

## Conclusion

Our study presented the largest series to date to demonstrate the significant survival benefit of ICI comparing to traditional chemotherapy in SD-UT. We also explored the prognostic disparity between SD-NSCLC and SD-UT under different settings, presented the clinicopathologic characteristics, and genomic features of the SD-UT and SD-NSCLC. ICI combination therapy may also be an effective strategy for perioperative treatment, but more study is needed.

## Supplementary Information

Below is the link to the electronic supplementary material.

Supplementary file1 Supplementary Fig. 1 Kaplan–Meier plots of overall survival in patients with thoracic SMARCA4-deficient undifferentiated tumor (SD-UT) and SMARCA4-deficient non-small cell lung cancer (SD-NSCLC) (TIF 3272 KB)

## Data Availability

The data that support the findings of this study are available on reasonable request from the corresponding author without compromising patients’ privacy.
