# Supplementary figures and images for: Promising efficacy of immune checkpoint inhibitor plus chemotherapy for thoracic SMARCA4-deficient undifferentiated tumor
Source: J Cancer Res Clin Oncol. 2023 Apr 28;149(11):8663–71. doi: 10.1007/s00432-023-04806-y (PMC10374696; doi:10.1007/s00432-023-04806-y)

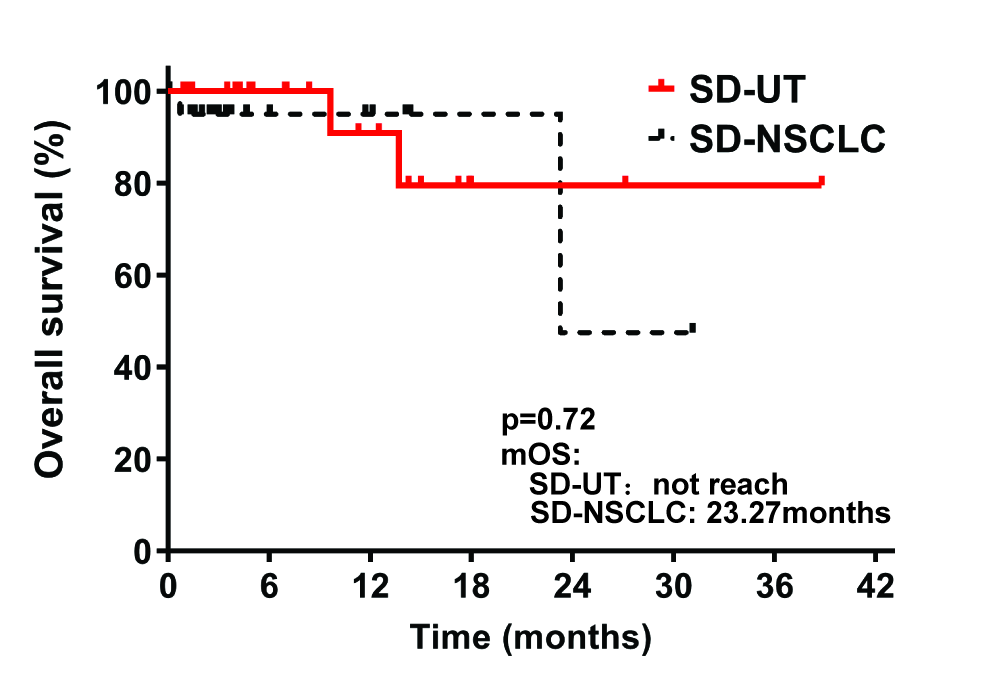

Supplement: Supplementary file 1 — Supplementary file1 Supplementary Fig. 1 Kaplan–Meier plots of overall survival in patients with thoracic SMARCA4-deficient undifferentiated tumor (SD-UT) and SMARCA4-deficient non-small cell lung cancer (SD-NSCLC) (TIF 3272 KB) [file 432_2023_4806_MOESM1_ESM.tif]
